# Ruptured Sinus of Valsalva Aneurysm: Silent and Rare Cause of Heart Failure in Young Adults

**DOI:** 10.1016/j.jaccas.2025.104566

**Published:** 2025-08-06

**Authors:** Toyokazu Endo, Emily Metz, Joshua Crane, Rohan Samson, Stephanie Moore, Sarabjeet Suri, Sheng Fu, Mark S. Slaughter, Siddharth V. Pahwa

**Affiliations:** a Department of Cardiovascular and Thoracic Surgery, University of Louisville School of Medicine, Louisville, Kentucky, USA; b University of Louisville School of Medicine, Louisville, Kentucky, USA; c Division of Cardiovascular Medicine, University of Louisville School of Medicine, Louisville, Kentucky, USA

**Keywords:** acute heart failure, aorta, congenital heart defect, imaging

## Abstract

**Background:**

Sinus of Valsalva aneurysm is a rare condition that can cause acute heart failure once it develops a fistula.

**Case Summary:**

A previously healthy 28-year-old man without history of intravenous drug use initially presents with an acute onset of heart failure symptoms. Transthoracic echocardiogram transesophageal echocardiogram revealed an aorto-atrial fistula causing significant left-to-right shunting. Blood cultures were negative for any infectious process. The patient initially required critical care with inotropic support and aggressive diuresis before going to the operating room for definitive repair. Intraoperative findings were consistent with the right sinus of Valsalva windsock deformity.

**Discussion:**

Fistula formation from a ruptured sinus of Valsalva aneurysm is a rare cause of acute heart failure in young adults. However, all other causes should be ruled out, including endocarditis.

**Take-Home Message:**

In the setting of young, previously healthy adults, ruptured sinus of Valsalva is a rare but silent cause of acute-onset heart failure.

## Visual Summary

**Figure:**
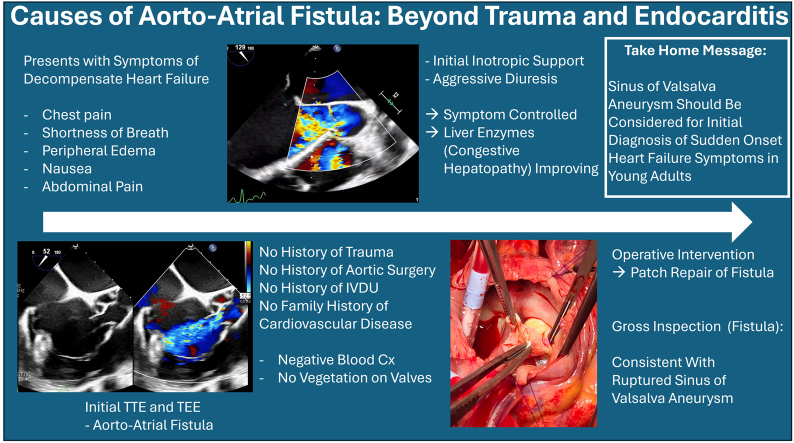

**Visual Sumary:**
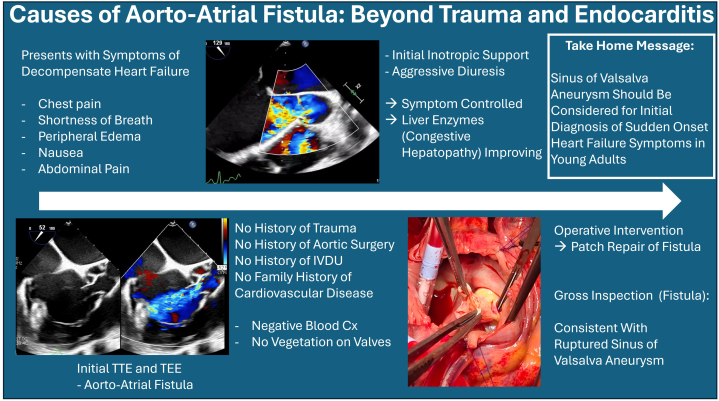
Ruptured Sinus of Valsalva Aneurysm

## History of Presentation

**Figure 1:**
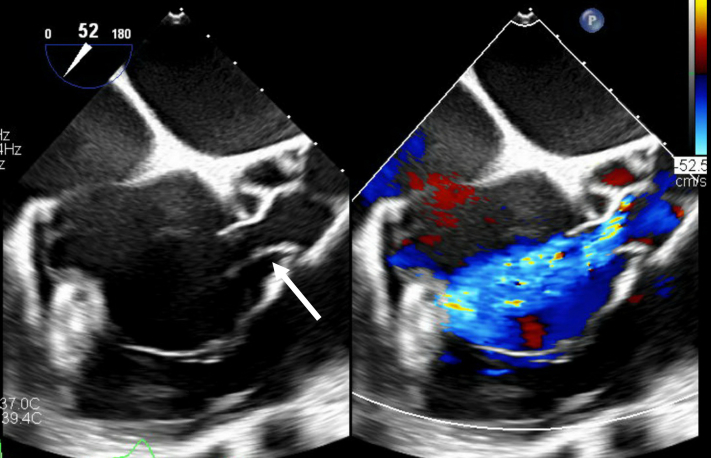
Initial Transesophageal Echocardiogram Showing Aorto-Atrial Fistula Open arrow indicates the aorto-atrial fistula.

The patient is a 28-year-old man who presented from an outside hospital for concerns of a fistula between the aortic valve and right atrium (Figure 1). On his initial presentation to the outside hospital, he reported chest pain, shortness of breath, bilateral lower extremity edema, and right-sided abdominal pain. He had also noted decreased appetite and nausea for 2 weeks prior. Laboratory results from the outside hospital initially showed creatinine of 1.2 mg/dL, sodium 134 mEq/L, potassium 4 mEq/L, aspartate aminotransferase 643 U/L, alanine aminotransferase 643 U/L, pro B-type natriuertic peptide 6004 pg/mL, D-dimer >10,000 mcg/mL. Venous Doppler was negative for deep vein thrombosis and computed tomography (CT) chest was negative for pulmonary embolism. CT abdomen/pelvis showed hepatomegaly with questionable hepatitis. Right upper quadrant ultrasound revealed hepatomegaly, steatosis, and ascites.

**Take-Home Messages**

• Aorto-atrial fistula is rare but can lead to significant left-to-right shunting, necessitating aggressive symptomatic management before proceeding with surgical repair.
• A ruptured sinus of Valsalva aneurysm should be considered as a differential diagnosis for sudden-onset heart failure in young adults.

The patient was transferred to our facility for a higher level of care due to concerns of heart failure and possible hepatitis. Vital signs were as follows: temperature of 95.9 °F, heart rate of 82 beats/min, respiratory rate of 10 breaths/min, blood pressure of 115/41 mm Hg, with mean arterial pressure of 62 mm Hg. oxygen saturation was 98% on room air. The patient was neurologically intact but drowsy and unable to answer questions. Cardiovascular examination revealed positive S_1_ and S_2_, regular, rate, and rhythm without murmur or jugular vein distention. Abdomen was soft, nontender, and moderately distended. A 2+ bilateral lower extremity pitting edema was noted. Radial and dorsalis pedis pulses 2+ bilaterally were noted. Repeat laboratory results at our center revealed creatinine of 2.65 mg/dL, sodium of 124 mEq/L, potassium of 5.2 mEq/L, aspartate aminotransferase of 623 U/L, alanine aminotransferase of 733 U/L, total bilirubin of 4.9 mg/dL, hemoglobin of 13 g/dL, platelet of 78 10^9^/µL, international normalized ratio of 3.82, and B-type natriuretic peptide of 2418 pg/mL. Blood cultures were negative; however, he was started on empirical coverage with cefepime, metronidazole, and vancomycin.

The patient was admitted to the critical care unit for further observation. At this time, cardiology, nephrology, gastroenterology, general surgery, and cardiovascular surgery were all consulted to collaboratively manage the patient's condition.

## Past Medical History

The patient's past medical history includes poorly controlled hypothyroidism managed with levothyroxine and obesity (BMI: 35.3 kg/m^2^). Social history is significant for homelessness, and he denied any prior history of intravenous drug use. The patient denied any history of surgical procedures and any family history of cardiovascular disease or malignancies.

## Differential Diagnosis

At the time of presentation, the patient was in acute decompensated heart failure with an unknown origin of his aorto-atrial fistula. There was no family history of cardiovascular disease, and there was no history of prior episodes of chest pain, or shortness of breath. Given that he had no prior history of aortic surgery, nor recent trauma in the area, these causes for fistula formation were ruled out. Thus, the differential diagnosis was narrowed down to possible endocarditis or ascending aortic aneurysm/sinus of Valsalva aneurysm.

In terms of the elevated liver enzymes and hepatomegaly noted on his CT scan from the outside hospital, hepatitis was initially considered as the potential cause. However, the significant left-to-right shunting causing congestive hepatopathy was more likely.

## Investigations

The patient's history and blood work was sufficient to rule out infective endocarditis as the cause of the fistula formation. The patient denied any history of prior intra venous drug use, and blood cultures were negative for any infectious process. Furthermore, there was no history that would suggest a remote history of rheumatic or scarlet fever. The transthoracic echocardiogram also did not show any evidence of vegetation on the tricuspid leaflets or on the aortic valve. In addition, liver biopsy was consistent with congestive hepatopathy. Given these findings, the fistula formation was likely due to the rupture of the sinus of Valsalva aneurysm.

## Management (Medical/Interventions)

The patient was managed for his decompensated heart failure with aggressive diuresis. Given his elevated liver enzymes, there was sufficient evidence to suggest that the left-to-right shunt had caused congestive hepatopathy. Over the course of 7 days in the intensive care unit (ICU), the patient's symptoms slowly improved with improvement in his respiratory status, improvement in lower extremity edema, and downtrend of his liver enzymes. After 19 days of aggressive diuresis to manage his heart failure symptoms, the patient was taken to the operating room for a definitive repair of his aorto-atrial fistula. Additionally, the patient underwent 11 days of inotropic therapy before surgery.

**Figure 2:**
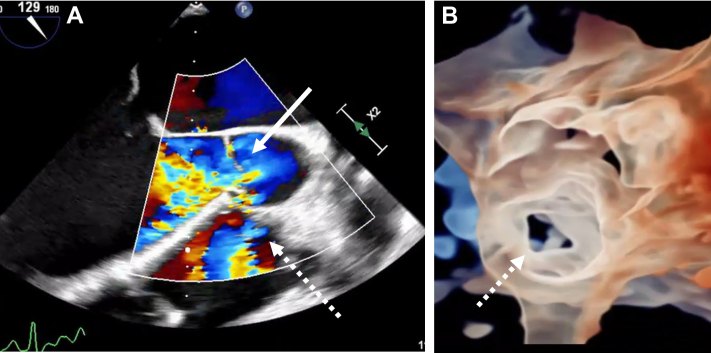
Intraoperative Transesophageal Echocardiogram Intraoperative transesophageal echocardiogram showed significant aortic insufficiency (open arrow), and an aorto-atrial fistula originates from the right sinus of Valsalva into the right atrium (dotted arrow). (A) Three-dimensional reconstructed image of the aortic valve also demonstrates the origin of the fistula (B).

**Figure 3:**
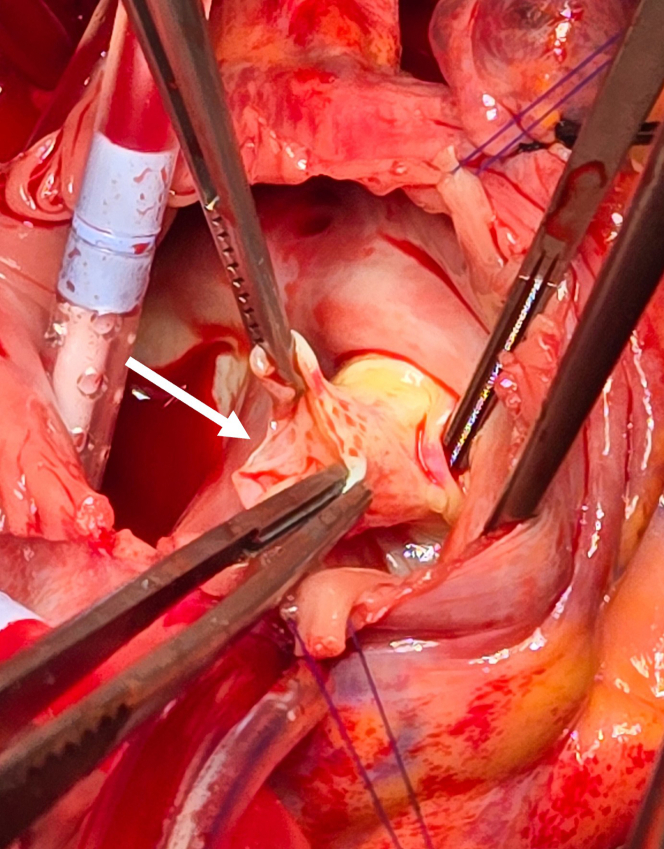
Intraoperative Finding of Windsock Deformity The aorto-atrial fistula is visualized from an opening created in the right atrium. The fistula (open arrow) is consistent with windsock deformity caused by rupture of the right sinus of Valsalva aneurysm.

In the operating room, the patient underwent a transesophageal echocardiogram. The aorto-atrial fistula appeared to be originating from the right coronary sinus of the ascending aorta (Figure 2, Video 1). The left ventricle was severely dilated, with global hypokinesis with ejection fraction (EF) of approximately 25% to 35%. In addition, there was concomitant severe aortic regurgitation and mild tricuspid and mitral valve regurgitation. The aortic regurgitation was likely due to the pressure difference from the left-to-right shunt that caused the right coronary cusp of the aortic valve to not fully close. After placing the patient on cardiopulmonary bypass and inducing cardiac arrest, the ascending aorta was transected proximal to the aortic cross clamp. The fistula was between the right coronary cusp of the sinus of Valsalva and the right atrium. The right atrium was opened to properly visualize the defect and prepare for our repair. Given the windsock deformity, it was determined that the likely cause of the fistula formation was the rupture of the sinus of Valsalva aneurysm (Figure 3). The fistula was repaired using a bovine pericardial patch from within the aorta and was reinforced from the right atrium. After we were satisfied with the repair, the right atrium and aorta were reapproximated. Before coming off cardiopulmonary bypass, transesophageal echocardiogram revealed no shunt at the site of repair without evidence of tricuspid regurgitation or aortic insufficiency. However, there was diffuse left ventricular hypokinesis with an EF of 10% to 15%. The reduction in EF is likely due to myocardial stunning postcardiopulmonary bypass. Thus, the patient was transferred to the ICU for postoperative support and inotropes.

## Outcome and Follow-Up

In the ICU, the patient required 2 days of inotropic support before being transferred to the floor, and diuretics were also continued. He was extubated within the ICU on postoperative day 1, and was able to come off all inotropes on postoperative day 4. His postoperative course was uneventful and he made a full recovery and was discharged from the hospital on postoperative day 8, after a total of 27 days in the hospital. Despite continued medical treatment, his EF at the time of discharge remained at 10% to 15%, but without dyspnea or signs of peripheral edema. He was started on guideline-directed medical therapy for his new heart failure with reduced EF. His follow-up transthoracic echocardiogram at 3 months did show a slight improvement of left ventricular function with an EF of 20% to 25%.

## Discussion

We describe an extremely rare case of aorto-atrial fistula that most likely originated from a right sinus of Valsalva aneurysm. The severe left-to-right shunt caused a sudden onset of decompensated heart failure that required aggressive medical management to stabilize the patient to surgical repair. The incidence of aorto-atrial fistula is extremely rare, with a reported incidence of approximately 1% to 2% of all aorto-cardiac fistulas.^1^ In the literature, the cause of aorto-atrial fistula is due to trauma, endocarditis, prior aortic surgeries, and sinus of Valsalva aneurysms.^2^ Of these, sinus of Valsalva aneurysm is even more rare, with a reported incidence in the general population of 0.15% among all patients undergoing open heart surgery.^3^ Typically, these present in young, previously healthy adults and are more common in the Asian population.^4^ The cause of such aneurysm is not well known given the rarity. However, it can be attributed to acquired risk factors such as inflammation and weakness around the aortic wall or congenital weakness.^5^^,^^6^ Although the aneurysm can happen in any of the 3 sinuses, the right coronary sinus is the most common (70%-90%), followed by the noncoronary sinus (10%-25%) and then the left sinus (2%-5%).^7^ Thus, a fistula formation due to the rupture of the aneurysm has been classified according to the modified Sakakibara classification, with our patient classified as IIIa (penetration and rupture into right atrium adjacent to or at tricuspid annulus).^8^

As with our patient, aorto-atrial fistula can cause heart failure due to severe left-to-right shunt, with associated dyspnea, pedal edema, and congestive hepatopathy.^9^ The management of heart failure initially is supportive but, in all cases, requires a definitive surgical repair. However, the timing of such repair is dependent on the etiology of the fistula. In the case of infective endocarditis, the decision to repair requires a multidisciplinary approach to balance the risks associated with graft infection, microemboli, or taking an unstable patient to the operating room.^10^ In our patient, there was no concern for an infectious process. Thus, the timing of the operation depended on optimizing heart failure symptoms. The repair can include a simple fistula closure primarily, patch repair, or aortic root replacement, all of which we do not have sufficient follow-up data or numbers to report which of these is the best approach.^8^

## Conclusions

Aorto-atrial fistula resulting from sinus of Valsalva aneurysm is very rare. Nonetheless, it should be considered in the initial differential diagnosis for sudden heart failure symptoms in patients who show no signs of endocarditis or trauma.

## Funding Support and Author Disclosures

The authors have reported that they have no relationships relevant to the contents of this paper to disclose.

## Appendix

Video 1 Intraoperative Transesophageal Echocardiogram Aorto-atrial fistula originating from the right sinus of Valsalva (open arrow).
